# Ethical Issues in Consent for the Reuse of Data in Health Data Platforms

**DOI:** 10.1007/s11948-021-00282-0

**Published:** 2021-02-04

**Authors:** Alex McKeown, Miranda Mourby, Paul Harrison, Sophie Walker, Mark Sheehan, Ilina Singh

**Affiliations:** 1 Department of Psychiatry, Wellcome Centre for Ethics and Humanities, Warneford Hospital, University of Oxford, Oxford, OX3 7JX UK; 2 Centre for Health, Law and Emerging Technologies (HeLEX), University of Oxford, Oxford, UK; 3 Department of Psyhiatry, Oxford Health NHS Foundation Trust, University of Oxford, Oxford, UK; 4 Department of Psychiatry, University of Oxford, Oxford, UK; 5 Ethox, Wellcome Centre for Ethics and Humanities, University of Oxford, Oxford, UK; 6 Department of Psychiatry, Wellcome Centre for Ethics and Humanities, University of Oxford, Oxford, UK

**Keywords:** Big data, Ethics, Machine learning, Consent, Health data platforms, Social contract

## Abstract

Data platforms represent a new paradigm for carrying out health research. In the platform model, datasets are pooled for remote access and analysis, so novel insights for developing better stratified and/or personalised medicine approaches can be derived from their integration. If the integration of diverse datasets enables development of more accurate risk indicators, prognostic factors, or better treatments and interventions, this obviates the need for the sharing and reuse of data; and a platform-based approach is an appropriate model for facilitating this. Platform-based approaches thus require new thinking about consent. Here we defend an approach to meeting this challenge within the data platform model, grounded in: the notion of ‘reasonable expectations’ for the reuse of data; Waldron’s account of ‘integrity’ as a heuristic for managing disagreement about the ethical permissibility of the approach; and the element of the social contract that emphasises the importance of public engagement in embedding new norms of research consistent with changing technological realities. While a social contract approach may sound appealing, however, it is incoherent in the context at hand. We defend a way forward guided by that part of the social contract which requires public approval for the proposal and argue that we have moral reasons to endorse a wider presumption of data reuse. However, we show that the relationship in question is not recognisably contractual and that the social contract approach is therefore misleading in this context. We conclude stating four requirements on which the legitimacy of our proposal rests.

## Introduction

Data platforms created to embody the principles of ‘open science’ (Choudhury et al. 2014), represent a new paradigm for carrying out research. Examples of these include Dementia Platform UK (DPUK); and the MQ Adolescent Data Platform in the UK; pan-European initiatives such as the Electronic Health Records Systems for Clinical Research (EHR4CR) and the NIH All of Us Research Programme in the USA. Data platforms offer a way of doing (health) research that follows from the possibilities purportedly offered by machine learning and predictive analytics (Hafen 2019). In the platform model, cohorts are shared with and between those institutions that hold the data for analysis and research so that novel insights useful for developing better stratified and/or personalised medicine approaches can be derived from their integration (McIntosh et al. 2016).

The rationale underlying big data-driven healthcare is that linkage and integration of datasets pertaining to a wide variety of health indices will be able to provide an increasingly granular understanding of how biological, environmental, and social (Allen et al. 2014; Fisher and Baum 2010) factors interact either to influence health, illness, and disease (Marmot et al. 2012) or to produce correlations that are useful for making predictions about current and future health states. The approach frequently employs algorithmic prediction (Mittelstadt and Floridi 2016) enabled by advances in machine learning, which has the capacity to generate new knowledge more quickly than traditional scientific approaches, and reduces human bias in the collection and analysis of data. It has been claimed that the predictive capability of these innovations can be equal to conventional statistical and epidemiological means (Prainsack 2018). It has also been claimed that given the current technological trajectory and anticipated advances in these technologies, machine learning methods may enhance (Ghassemi et al. 2015) and eventually possibly exceed (Nielsen et al. 2019), human analytic capacities.

This purported eventual capacity is a central plank of the argument for the ethical legitimacy of machine learning techniques being applied to big data both generally and in the specific context of health data platforms. The increasing proliferation of these techniques is contributing to the establishment of a new health data infrastructure. Since the claim is that it may reveal novel and associations, machine learning can be used to underwrite the justification for reuse, as it keeps open the possibility that one’s data may have epistemic value in the future in ways that could otherwise not have been predicted.

It is important to note here, however, that such claims have not been universally accepted as accurate, as scepticism has been expressed about the fundamental limitations of machine learning to replicate, and therefore surpass in sophistication, the reasoning capacities of human beings. This claim has been robustly defended by Davis et al. (2013), Davis and Marcus (2015), Marcus et al. (2020), Marcus (2018). Their argument relies on the premise that the disembodied, uncoupled, non-perceptual nature of machine, rather than human, learning, limits its capacity to make the kind of lateral associations and leaps in comprehension that are a characteristic ability of human intelligence, drawing as the latter does on a palette of information sources more varied than the purely statistical operations that machines can perform. As such, if this argument is correct, failure to take into account the panoply of extra reasoning tasks that characterise human cognition when attempting to build machine learning models that can surpass it will only lead to an impoverished form of ‘intelligence’ that is similarly liable to error as humans, albeit for different reasons. For example, Marcus et al. (2020, p. 50) argues that:

Deep learning has—remarkably—largely achieved what it has achieved without…anything that looks like explicit modules for physical reasoning, psychological reasoning and so forth. But it is a fallacy to suppose that what worked reasonably well for domains such as speech recognition and object labeling—which largely revolve around classification—will necessarily work reliably for language understanding and higher-level reasoning. A number of language benchmarks have been beaten, to be sure, but something profound is still missing. Current deep learning systems can learn endless correlations between arbitrary bits of information, but still go no further; they fail to represent the richness of the world, and lack even any understanding that an external world exists at all.

There are also perhaps more moderate concerns that one might have here. We might, in a less extreme way, just think that a good deal of caution is required when assessing the overall benefits that these kinds of new technologies will bring—we may for instance be confident that will bring some benefit in some contexts but that the complete, transformative nature of the effect is yet to be clearly demonstrated. For example, the use of machine learning methods in cancer research to develop predictive models to improve our understanding of cancer progression is now well evidenced and translated into vastly improved treatment outcomes (Kourou et al. 2015). However, the use of machine learning applied to big data in Alzheimer’s disease research has yet to yield such fruit, and patient treatment has barely progressed in 20 years (Ienca et al. 2018). As a result, cancer patients may be less opposed to the reuse of their health data for research than dementia patients, but different rates of progress should not constitute grounds for dismissing the *potential* benefit of big data science overall.

Given this range of concerns, it is important to treat claims about the power of machine learning for radically accelerating understanding of the determinants of health, disease, and illness with caution in what follows. However, assuming we regard such claims as conditional and provisional, then *if* the integration of diverse datasets through sharing and reuse *does* enable the development of more accurate risk indicators, prognostic factors, or better treatments and interventions, this obviates the need for doing it; and a platform-based approach is an appropriate model for facilitating this. Platform-based approaches thus require new thinking about consent. We contend that two structural conditions obtain which compromise traditional approaches:

- In platform-based health data research, reuse and sharing of data by researchers granted access to these data is inevitable and necessary.
- Since a big data, machine learning approach is predicated on the value of its capacity to reveal novel findings and causal relationships beyond those that are predictable through conventional means, it follows from this unpredictability that the standard account of prospectively informed consent may be inadequate.

We suggest that these two conditions should be considered as fundamental practical principles of any approach to negotiating consent-related ethical dilemmas that arise in health data platforms. However, we also suggest that that these conditions are manageable. In what follows we defend an approach to managing them. This approach is grounded in three components: (1) the notion of ‘reasonable expectations’ for the reuse of data; (2) Waldron’s (1999) account of ‘integrity’ as a heuristic for managing disagreement about the ethical permissibility of the approach and; (3) the element of social contract approaches that emphasises the importance of public engagement in embedding new norms of research consistent with changing technological realities. In this paper, therefore, we argue for a normative presumption of health data reuse for research in data platforms, and we partially endorse the concept of a social contract in support of our argument. On the basis of the analysis which follows, we conclude by stating four requirements on which the legitimacy of our proposal rests.

## Consent for Reuse

Traditional models of informed consent may be ill suited to big data projects, because these tools were conceived in the context of conventional clinical research such as clinical trials, which are not concerned with the evolving applications and innovative research designs of big data research (Ienca et al. 2018). A hallmark of the claimed effectiveness of the big data analytic approach is its ability to make novel predictive associations about health, illness, and disease that can match, and may in future surpass, conventional human means (Michael and Miller 2013; Schadt 2012). Assuming the veracity of claims about such effectiveness, the possibility of unanticipated opportunities for future research is therefore a necessary feature of the justification for the use of this approach (Nickel 2019). Indeed, since unpredicted or unexpected findings are precisely what are hoped for, the question is pertinent as to whether, when, and how consenting to the *re*use of data for new research purposes should be managed, including instances where research might yield findings relevant to the health of the participant and about which they may, or may not, wish to be informed (Bishop 2009; Otten et al. 2015; Yardley et al. 2014). This issue has already attracted attention in the bioethics literature (Grady et al. 2015; Thompson and McNamee 2017; Zwitter 2014; Mcneely and Hahm 2014).

The consent for reuse issue is significant for three reasons in both the general context of health research and the specific context of where this is carried out via a data platform. First, understanding the aetiology of diseases requires their study longitudinally (Floridi 2012; Swan 2013). Second, the apparent power of big data analytics derives from its ability to make novel predictive inferences across datasets about the interactions of disparate risk factors (Kitchin 2014; Vayena et al. 2015). Third, this iterative novelty limits what can be communicated to participants about the purposes for which their data may be used (Otten et al. 2015). Illes and Chin (2008) remind us that what is foundational in the context of reuse is the welfare of research participants, which could be compromised if procedures concerning their personal information are not adequate or properly observed (Fiske and Hauser 2014; Zook et al. 2017). The harms, as well as the benefits, that might derive from data science research are unpredictable (Metcalf and Crawford 2016). Given this, it is unclear how the paramount condition of participant welfare is to be ensured. There is no overall consensus about how to define optimal participant welfare, or how consent for the reuse of data should be managed (D'Abramo 2015). However, several strategies have been advanced (Porteri et al. 2014).

In a ‘blanket’ consent model (Simon et al. 2011), such as is employed in the All of Us programme,^1^ participants agree in advance for their data to be used in any future research considered appropriate and relevant by those holding the data. This has the advantage of maximising the research uses to which data can be put, but the disadvantage of failing to inform the donor of the nature of the research. A more clearly proscribed iteration of this is found in ‘broad’ consent, where permission is sought for a range of uses but not assumed for all purposes and is constrained, for example by the area of research, or by governance conditions stipulated by owners of the cohorts or custodians of the platform,^2^ by which researchers are obliged to abide. This model shares many of the advantages of the blanket consent model, although it too has potential drawbacks. For example, a study may be proposed which requires consent from individuals at high risk of developing a particular condition for the reuse of their data. In this instance, consent would be contingent on informing these individuals of their high-risk status. Although this satisfies the traditional standard of consent that it is sought for a specific purpose, it also presents its own ethical challenges, given the potential distress that such a disclosure may cause and its potential implications for the patients’ right not to know where genetic risk is involved.

Extending this approach, a third alternative is ‘dynamic’ consent(Mostert et al. 2015) and is similar to the traditional model, insofar as consent is sought on a case-by-case basis, although in this instance for reuse of data for each specific purpose (Goodman et al. 2016), rather than for its initial use. This has the advantage of meeting the apparent ‘gold standard’ (Thompson and McNamee 2017) of consent to the extent that permission is sought from participants to ‘opt-in’ for each new use of their data in a particular study. However, it has the drawback that this model is not likely to be suitable in instances where data subjects are unwilling or unable to have ongoing engagement with a digital research interface (which may be true of various vulnerable or harder to reach groups, or even of a general population less interested in the research) (Teare et al. 2017). Moreover, there are serious questions about whether this depiction of the ‘gold standard’ is ethically appropriate given the range and variety of choices which we routinely and acceptably make (Sheehan 2011). Additionally, some people may refuse to allow their use of their samples for particular uses, and some individuals may be or become uncontactable. As such, this approach may limit the scale, value, and validity of studies carried out which employ it (Walker et al. 2019). The complexity of this challenge is amplified in international research platforms which draw on data from different legal jurisdictions, for example in the EHR4CR programme,^3^ since established protocols for conditions of reuse in these jurisdictions may not be uniform.

A fourth option, therefore—and which some would describe as a form of dynamic consent—is ‘meta’ consent (Ploug and Holm 2015). Under a meta-consent model, individuals would be able to choose how they prefer to provide consent—for example, whether they prefer a blanket or dynamic model for future uses. On the significant assumption that this model is distinct from the dynamic consent model (Sheehan et.al. 2019). this model, like dynamic consent, has the advantage of putting participants as much as possible in control of their data; however, it has been criticised for still failing to meet the gold standard of consent given that it does not circumvent the unknowability of potential future uses that is a function of a predictive analytic machine learning approach (Manson 2019).

Given the plurality of interests involved in these scenarios, there are likely to be differences of opinion as to which consent model is ethically and practically optimal. As Heeney and Kerr (2017) note, non-traditional models of consent that would enable easy reuse are typically favoured in the health science and policy arena, precisely because they can expand research in beneficial new ways. However, for the reasons outlined above, public preferences for these differs (Goodman et al. 2016; Simon et al. 2011; Sundby et al. 2019). As such, views of what is desirable and appropriate may differ between investigators and participants (Appelbaum et al. 2014).

Whatever the content of competing views might be, if traditional models of consent are inadequate for contemporary healthcare research, then a new and more satisfactory approach must be found. To develop an approach to consent that can make possible sustained uses of data for medical research on the basis of well-founded trust and confidence, a suggested way forward has been to establish a new ‘social contract’ (Desmond-Hellmann 2012; Lucassen et al. 2017; Horne et al. 2015; Vayena et al. 2016) that can overcome the difficulties presented by standard approaches to consent and agree with the public what counts as reasonable expectations for health data reuse.

The proposal appears rational, but little attention has so far been paid to the process of translating the apparent theoretical solution into practice. For reasons we explain in what follows, while a social contract approach sounds appealing in certain respects, it is less coherent when applied to the context at hand. Rather, we defend a way forward that upholds that element of the social contract which emphasises the importance of openness and democratic engagement in what is proposed, but which rejects the claim that the desired relationship between individuals, the institutions holding and using their data, and the state is accurately characterised as contractual in any way that is not misleading. We argue that the move to a presumption of the reuse of data is a proposal that the public would have reasons to endorse, and it is the legitimacy of these reasons that provide the normative force for the proposal and the legitimacy of seeking public assent to it. However, for reasons which we will unpack, it is misleading to characterise the relationships between the public and institutions of health and data governance as one which resembles a contract in any legal or conventional sense of the word.

## How Coherent is a Social Contract for the Reuse of Health Data?

One interpretation of health care, and by extension the health research required for being able to deliver it effectively, is that it should be conceived of as a common good^4^ (Prainsack 2018). In the contemporary context this interpretation is pertinent to the claim that the routine collection and reuse of large data sets might yield both better epidemiological understanding and more effective personalised treatments (D'Abramo 2015). If health research of this kind *is* a common good in view of the aggregation of these data yielding not only individual benefits but population-level, public health benefits, and *if* the wider unconsented reuse of data does yield these benefits, it can be argued that it is something in which we all *ought* to participate.

Assuming this characterisation of an obligation to permit the use and reuse of our data in research is coherent, a further argument can be made in favour of a *presumption* that we do so, for example by assenting to the reuse of our data for research unless we actively opt out by withdrawing our permission (Ballantyne and Schaefer 2018). Before considering the social contract in more detail, however, it is important to consider the different reasons individuals may have for opting out and, at least according to the argument we make, failing to meet their obligation to the improvement of health through research. Some of these reasons are better than others, and their legitimacy also turns on whether or not concerns about privacy and harm are well-founded.^5^ [.]

Much of the data held on health data platforms is de-identified and/or anonymised, and the potential scientific discoveries made by health data platforms are made on a population level, not on an individual level. It is therefore not possible to draw conclusions about an individual’s health status or provide personalised feedback from analyses to specific participants. However, if this is not made clear to participants, an individual may wish to withhold or withdraw consent for their data to be used in health research due to fear about what may be discovered from the analysis of their data, such as genetic risk profile for a disease. Many research participants do not want to know their risk status, but of course many people do so that they can redress this power imbalance and make informed medical and lifestyle choices to mitigate the disease risk. Consequently, an individual may wish to withhold consent because they are worried about their genetic risk profile being sold to an insurance company, the disclosure of which subsequently leads to an increase in the cost of health insurance or its denial altogether.

While it would be technically possible for malicious attempts to de-anonymise data to occur within health data platforms, making the above concerns reasonable ones, the risks can be managed with efficient regulatory and technical measures to ensure privacy-preserving techniques such as encryption and block chain are incorporated into the digital infrastructure (Ienca et al. 2018). The risk of data being sold for commercial or insurance purposes can be legislated against and thus managed by good governance. If the risk can be eliminated by making it illegal for health data platforms to sell data to insurance companies, then the justification for withholding consent is no longer reasonable. Of course, it is important to be careful about what the specific regulations are, but if this can be achieved such that perceived risks are blocked, then claims about such risk can be shown to be false, such that individuals no longer have well-founded reasons not to assent to the presumed reuse of their data.

There is, therefore, a distinction to be drawn between reasons based on genuine harms that can be addressed and protected independently by governance, oversight and legislation, and reasons based on personal preference which, at least according to the argument we make, are outweighed by the common good. The right for individuals to withdraw their data from use in research must be upheld in instances from the former category, where the grounds for doing so are legitimate, and the protection of this right partly underpins the four conditions under which our proposal is justified. An example to illustrate instances from the latter category, however, where the reasons given are not sufficient to justify withdrawal, might be an individual who is racist and wishes to withhold the reuse of their data for research which may yield benefits to the health of a particular ethnic minority. This is indeed a preference, but it does not count as a good reason to agree to the withholding of consent because (1) racism is wrong; and (2) it would not conduce to the public good to limit the scope of research which could help a particular group which is arbitrarily discriminated against. As such, because the request to withhold consent does not provide good reasons for doing so, we could coherently insist that research for ethnic minority groups should be protected by not agreeing to dissent on grounds of race.

This discussion of what counts as legitimate and illegitimate reasons for dissenting from approval for a widespread presumption of data reuse is relevant for what follows, and we will return to it in due course. However, it is important to flag the distinction at this point, because the legitimacy of the proposal of a wider presumption of date reuse depends on the elimination of justified reasons for individuals not to assent to it, such that data can be used in a way that yields optimal public benefit. As we indicated above, since what is at stake here is optimal public benefit balanced with appropriate respect for participants, in the context of health data reuse, even though we are considering the use of these data for research, it is, as Ballantyne and Schaefer (Ibid.) clarify, more accurate to consider this proposal as a matter of public health ethics than one of research ethics per se. This is because social contract-type proposals (notwithstanding that we dispute the accuracy of this characterisation) aim at justifications which for balance aggregate good with individual liberty, albeit by appealing to rational individual interests to secure it.

Assuming we accept that it is possible to discern legitimate and illegitimate reasons for withholding or withdrawing consent, then, it may appear to follow that to aim at the common good is necessarily to endorse a ‘social contract’ (Freeman 1990) as a means of research governance. Indeed, this is precisely the proposal that has been made in the health data research context (Desmond-Hellmann Ibid; Lucassen et al. Ibid; Horne et al. Ibid; Vayena et al. Ibid). However, for reasons we explain in what follows, this mischaracterises the relationship in question. Closely associated with Hobbes, Locke, Rousseau, and more recently, Rawls, the social contract has a long pedigree. Despite other differences between their accounts of the contract, what is consistent across them is the principle that a social contract would enshrine mutually beneficial social, legal, or ethical rules to which all members of society have good reason to assent. This is to say that a social contract is one in which agreement is given to a code of conduct governing a class of activities to which assent is subsequently assumed—as opposed to the ‘contract’ establish through consent being explicitly constructed for particular purposes in each specific instance—in view of the benefit that each individual would derive from doing so (Freeman 1990), and notwithstanding whatever other, inevitable, differences there might be between individuals (Rawls 1958).

The thrust of this argument is reflected in the position advanced by Lucassen et al. (2017, p. 3) who advocate a social contract for genomic medicine in the NHS predicated on an expanded presumption of the use and reuse of patients’ health data, given that:

…linking up of large data-systems containing personal identifiable data on a scale not previously necessary (or possible), is a prerequisite for success…genomics will provide both diagnoses and predictions and will affect patients, families, the general public in different ways over time.

The difficulties associated with securing satisfactory consent in the traditional sense in the big data context are formidable. Therefore, reconceptualising this dimension of research in terms of the contractual conditions by which rational individuals would agree to be bound may be one way to surmount the challenges to consent that the digital data paradigm presents. This is appealing, but it is vulnerable to several objections, five of which we outline here.

First, any ‘contract’ is an abstraction until its details are enumerated and open to scrutiny as a concrete proposal or policy with which individuals are invited to agree; and while one may find a contract model rationally persuasive as an abstraction, if it turns out that one disagrees with its terms when instantiated, one is less likely to endorse it (Thrasher and Vallier 2015). Second, this is important because *no actual* contract is an abstraction. All contracts have terms, and it is a contingent matter whether specific individuals do or do not agree to them (Gaus 2011). Third, a pluralistic society advocates that the diverse values and preferences of individuals be respected and upheld; and since we live in a pluralistic society, there is no guarantee that the precise terms—whatever they happen to be—of the contract, will be universally agreed upon (Muldoon 2017).

Fourth, and a modulation of the third point, the contract has no answer to more extreme consequences of pluralism, namely, instances where we are prepared to say that different views some other people hold are just unethical or wrong. In the same way that, for example, the moral impermissibility of racism is not a matter of individual opinion, perhaps we ought to have the courage of our collective convictions with respect to adopting a position on the consent for reuse question. This would consist in straightforwardly defending the normative claim that it is unethical to seek to prevent one’s personal data from being reused in health research (while stopping short of outlawing the revocation of data and consent, however), because the circumstances in which we find ourselves make this the optimally just approach.

Fifth, and most importantly, a social contract approach is vulnerable to the objection that the situations which it would be created to codify and enshrine in an agreement is not contractual but more resembles a *fait accompli* (Brassington 2014). This is to say that, unlike *actual* contracts, one does not have the choice to ‘opt out’ of a socio-technological milieu in which a presumption towards the reuse of data happens to be the optimal strategy for maximising the possible health gains from research. Honesty that this is the reality of the situation would be required for securing public assent to the kind of proposal we outline here, and it functions as one of the four principles which underwrites the case we are making. Given that we cannot straightforwardly ‘opt out’ in the context at hand in the way that we would from many other arrangements, in this respect the health research infrastructure is better understood as a phenomenon that has emerged organically in response to the kinds of needs, capacities, and values that humans tend to have, rather than a discrete and separate, purely technical institution that can be straightforwardly entered or left at will (Lloyd 1901). Given that from birth all people require medical services that involve health data collection and have little choice in the matter about those needs, the description of this relationship as one which is straightforwardly ‘contractual’ is not coherent, despite appearing so at first sight (Riley 1973). Notably, these issues track the distinction between the idea of a social contract as it is understood in the political philosophical tradition—as a way of justifying, in principle or hypothetically, antecedent existing kinds of social structures like the state with a range of coercive powers—and the simpler idea of an actual (legal) contract. An actual contract requires certain specific conditions and consents whereas hypothetical is more flexible but arguably less able to secure specific requirements.

It is true, of course, that one can both accept the justificatory role of a hypothetical contract and still object to a relaxation of the standard consent procedure towards a presumption of reuse. However, since one does not ask to be born and instead simply finds oneself in a particular set of historically contingent circumstances without first having given one’s approval for it, the comparison with an actual contract breaks down: the contract element acts as a fig leaf for something less voluntary, given that valid contracts are predicated on having been freely entered into according to terms set out and agreed beforehand. Given this fault in the analogy, we recommend a different approach which does not characterise the relationship in question as contractual, but nevertheless draws on that element of the social contract approach that must be retained for the relationship to have legitimacy.

Mittelstadt (2019) notes that the kinds of dilemmas which arise in the context of data-driven, machine learning-dependent health research strategies are contemporary instantiations of ancient dilemmas and as such are not likely to suddenly yield clear and unarguable moral certainties. In view of this, disagreement will persist even if we could somehow secure widespread assent to the envisaged contract. Of course, the social contract approach is valuable to the extent that, by reminding us that these social institutions operate at the level of trade-offs between the common good and individual liberties, it emphasises the moral importance of engaging society broadly and the public more specifically, in the implementation of the proposal being made (Freeman 2000; Rossi 2014). We endorse this component for delivering good and ethical research governance: that is, we endorse the ‘social’ aspect of the relationship without endorsing the idea that the form of this relationship is properly thought as contract-like. Nevertheless, we defend the claim that a presumption of the reuse of data *is* how we *ought* to conduct health research, and by extension, downstream care as a result, and that the normative force of this holds independently of whether or not one happens to agree with the proposal.

In the next section, we demonstrate this and defend our argument by drawing on Taylor and Wilson’s (2019) argument for the use of ‘reasonable expectations’ as an alternative basis to consent for the disclosure of health data; and Waldron’s (1999) account of ‘integrity’ as the central value required for justifying particular arrangements.

## Reasonable Expectations and Integrity

Public assent to a greater presumption of the reuse of data would require at least, in the first instance, the articulation of the arguments in favour of the proposal to those people who are uncomfortable with its implications, even though some proportion of these people will undoubtedly remain unpersuaded and persist in their objection. In this regard, Taylor and Wilson (Ibid, p. 459) acknowledge that *‘there is much to do’* with respect to social agreement about what expectations are considered acceptable regarding consent for new uses of personal health data. To meet this challenge they advocate *‘collaborative public reasoning’* about such expectations, for example in the form of citizens’ juries of the kind sponsored by the National Data Guardian (2018). These kinds of engagements are necessary for the democratic legitimacy of proposals such as the one we are considering here, and their importance is such that effective public engagement constitutes another of the four conditions on which the legitimacy of our proposal rests. It must be recognised, however, that efforts are time consuming and move at a slower pace than the relevant technologies requiring governance solutions.

Nevertheless, to the extent that the social contract can be any kind of useful guide, what they have in their favour is the requirement that a balanced articulation of the situation is proposed for the seeking of assent. Even if the social contract model is ultimately redundant in view of the ‘contract’ element of it mischaracterising the relationship in question, it will still be important to give an account of why, in this case, a presumption towards the reuse of one’s data is rational and *all-things-considered* the optimal normative proposal. Given that disagreement is inevitable, and even if it cannot be fully overcome, Waldron argues in situations such as this that a just outcome can be reached if the process is carried out with what he defines as *‘integrity’* (1999, p. 195), where this is understood as ‘the elaboration of respectful procedures for settling on social action despite the stand-off’*.*

Taylor and Wilson (Ibid, p. 451) argue for a model of reuse justified on the basis that it would be ‘reasonable’ to expect it to occur, based on the evolution of the common law of confidence to suggest that a patient only has a right of privacy vis-à-vis those parties whom they have *not* understood and accepted will have access to their medical information.^6^ They argue that this evolution in the law aligns with the new, multilateral nature of contemporary data-driven healthcare. Crucially, although this deemed acceptance is dependent on all circumstances of the case,^7^ unlike consent it is not dependent on the subjective mindset of the individual. Regardless of whether a particular individual expected the data sharing, the question is whether a reasonable person with ordinary sensibilities in their position would expect their identifiable information to be shared (for example, for clinical audit or for research).^8^ This creates a more stable, objective basis for sharing information.

While privacy is a broad concept, and difficult to define categorically (Laurie 2002), it is often associated with norms of exclusivity or control (Nissenbaum 2004; Taylor 2012). In other words, information is deemed private if a reasonable, ordinary person (the normative benchmark) would consider it to be so. The norms that underpin these ‘reasonable’ expectations are not static and are liable to change with societal shifts and public discourse. Moreover, it does not follow from reuse being a reasonable expectation in the sense that one would not be *surprised* to find out that it happened, that the proposal of a presumption of reuse is reasonable in the sense of it being what *ought* to be the case. For example, just because there might be a ‘reasonable expectation’ in some countries that if I am convicted of a murder, I am *likely* to be put to death by the State, the expectation is agnostic about whether or not capital punishment itself is reasonable in the sense of being morally *justifiable*. In this regard there is a distinction that must be made explicit between reasonableness in the *descriptive or statistical* sense (what people actually take to be reasonable), and in the *evaluative or normative* sense (what is justifiably or ought to be taken to be reasonable), to avoid confusion between the two ways in which the term may be understood (Buchanan and Keohane 2006). In the context of a proposal of a greater presumption of the reuse of health data, therefore, the legitimacy of the proposal—this to say, the reasonableness of the proposal in the normative sense—depends on a robust and explicit case being made in favour of it. This case is grounded in three salient circumstantial considerations.

The first two of these considerations are the conditions outlined earlier pertaining to data platforms, namely that: (1) optimising the benefit from data-driven healthcare requires the sharing and reuse of data; and (2) the reach of machine learning to uncover otherwise unpredictable associations between, and by extension uses for, health data makes necessary a reassessment of consent in this context. The third consideration is that, as Taylor and Wilson (Ibid, p. 437) note, UK healthcare institutions play a fundamental role in establishing reasonable expectations for reuse of data, and have already *‘committed to the principle of collect once, use many times’* on the basis of the 2014 memorandum of understanding established between NHS England and the General Pharmaceutical Council. Crucially, commitment to the principle of reuse after collection follows from the recognition—reflected in the first two considerations raised—that the imposition of too many restrictions on the sharing of data for reuse ‘sits uneasily with…data flows necessary to deliver care in the context of a modern healthcare system’ (Taylor and Wilson Ibid, p. 434).

On the basis of these considerations it is possible to argue for the legitimacy of a presumption of the wider reuse of data than currently permitted by standard models of consent, if we hold that we should wherever possible seek to optimise the potential population health gains using the technological means available. Key to the legitimacy of such a proposal is the integrity of the claim that a broader conception of the conditions under which data can be reused is necessary, and by extension *should be expected* in the service of the relevant health goals. Taylor and Wilson (Ibid, p. 439) summarise:

If persons would not be surprised to learn that information had been used for a particular purpose, even if they did not consider themselves to have positively signalled consent to that use, then we may protect social licence in processing for diverse purposes without overburdening the consent process.

However, even if we accept the argument so far and agree that part of the legitimacy for a given use follows from whether or not we would be surprised to discover that it had been used for that purpose, it is fair to note a valid objection, articulated in Waldron’s analysis of the complexities of public consensus-seeking such as this. Although the response to this objection does not completely extinguish strictly moral theoretical concerns, there are reasons to think that legitimacy can nevertheless be achieved in support of the kind of proposal we are making.

The objection is that the nature of moral discourse is such that disagreement can never be fully extinguished. There is no way to *prove*, in the way that is more readily available in the sciences, that a statement about the right course of action is true. Since competing moral positions have competing standards of justification, so ‘they share virtually nothing in the way of an epistemology or a method with which these disagreements might be approached’ (Waldron 1999, p. 177). Consequently, even universal consensus would be insufficient for such proof, given that in the absence of a more fundamental epistemology or method, ‘The prospect of majority support adds nothing to the reasons in favour’ (Waldron Ibid, p. 197) of whatever is being proposed.

In response here it is consistent to concede that progress towards a new norm of research in which presumptions of non-disclosure pertaining to the reuse of data are relaxed may indeed be slow, incremental, and imperfect: indeed, it is just a fact about trust that it takes time to build and requires effort and resources to do so (Kraft et al. 2018), particularly in the area of educating the public about how this kind of new platform-led science is done and why it is important for progress. The pace at which the public builds trust towards this new norm may parallel the pace at which the returns of these greater gains from open science translate into improved treatment and care for patients. This is to say that the more visible the benefit, the greater the likely acceptability.

In the context of growing distrust of the way companies such as social media platforms mishandle personal data, and in view of legitimate public concerns about instances in which the secondary use of data has been inadequately governed,^9^ it is also vital for the public legitimacy of greater reuse of data that the institutions handling the data can be relied on to devise and deliver the research responsibly, such that trust in these institutions is not undermined. As Sheehan et al. (2019, p. 11) remind us:

…research is designed by researchers and institutions which are responsible to society. This involves the use of appropriate expertise and authority to construct and conduct research that produces public benefit.

With this in mind, we can assert that if the goals of health improvement being sought are sufficiently valuable at the individual and societal levels then the objections laid out do not undermine the reasons for attempting to make such progress towards those goals and definitively making the case for it, assuming that the institutions handling the data are trustworthy and can be relied on not to abuse the trust placed in them by the public. It might be objected here that this claim is question-begging, since it is precisely the trustworthiness that cannot be assumed. However, even if it cannot be *assumed*, trustworthiness can be secured using rigorous governance and the application of appropriate regulations, devised according to, for example, the kind of weighing outlined earlier with respect to the difference between legitimate and illegitimate reasons for withholding consent. Further, the introduction of updated legislative standards such as the General Data Protection Regulation (EU 2016) to hold these institutions to account if they misuse data may help to strengthen their relationships with the public. Importantly, the move from a focus on ‘trust’ to a focus on ‘trustworthiness’ is key for the suggestions here. Being trustworthy is within the power of institutions and their governance in a way that being trusted is not (Sheehan et al. 2020): we should endeavour to establish institutions that operate and are governed in a trustworthy way and this will allow those individuals who are able, to have firm grounds on which to trust. As such, trustworthiness constitutes another of the four conditions which legitimates our proposal.

Taylor and Wilson (Ibid, p. 459) point out that since changes to norms of consent will inevitably raise numerous questions and procedural ethical obstacles, so ‘Any extension would need to be cautious, gradual and firmly evidence-based’*.*^10^ Sharing identifiable health information without engaging the right to privacy (they argue) requires careful attention to adequate respect for individuals’ autonomy (Ibid, p. 458). As such, we are not suggesting that a presumption in favour of re-using health data for research would replace the context and attribute sensitivity of reasonable expectations^11^; it would merely be a normative influence which could help to shape this expectation in favour of reuse for research. However, this does not undermine the claim that integrity in the process according to which the proposal is implemented must be possible, since insofar as integrity is ‘a response to variety and dissonance’ (Waldron Ibid, p. 192), there would be no need for it in a world in which agreement on justice were total and unanimous. On the basis of this analysis, we therefore acknowledge that changing societal attitudes towards a presumption of the reuse of data in the context of contemporary health care and longitudinal research may be gradual: here, of course, is the shift between what is ‘normatively’ reasonable and what is ‘descriptively’ reasonable.

Nevertheless, given the potential health gains available from doing so, it is reasonable to unambiguously make the argument that enabling research to be done according to this new norm is *all-things-considered* justifiable and worthwhile. Moreover, norms *can* and *do* change; notwithstanding deep-rooted worries about the inconclusiveness of moral realism or any particular moral theory, these concerns are residual in this case if there are historical instances which we are prepared to commit to as those in which genuine moral progress has been achieved through changing societal norms. If we *do* believe that moral progress has been possible in spite of these insoluble philosophical challenges, and if we accept the argument laid out here, then we should commit to the possibility that it is achievable, even if incrementally, in this case.

## Conclusion

In this paper we have advocated for a normative presumption of health data reuse for research in data platforms, and partially endorses the concept of a social contract in support of this claim. We have defended this position for several reasons. Democratic legitimacy of big data-driven healthcare and research in general, and via the platform paradigm in particular, will be contingent on widespread assent to a norm which presumes the probable reuse of one’s data for purposes as yet unspecified. In the UK context, the NHS could play an important role in influencing public acceptance, given its particular national significance. Prior to the eventual achievement of this acceptance, however, our normative claim is that the proposal embodies the ethically correct course of action with respect to norms of health research given the situation in which we find ourselves with respect to the technologies available that can be applied to it. Given that this situation is, literally, one in which we find ourselves without having been asked for our permission and without having voluntarily entered it on that basis, we should resist characterising the relationship between individuals, the state, and health research institutions as one that is contractual. The proposal is, we suggest, the right one and should be defended on its merits in the first instance, social contract or no social contract.

Nevertheless, public legitimacy for the proposal *is* essential. Even though our normative claim is that there should be a presumption of data reuse in research, given that we live in a democracy, it is vital to secure assent to this new norm for optimising the benefits potentially derivable from it in future, given that trust in the institutions responsible will be required to achieve them, and this may be difficult. The public acceptability of these conditions *as norms* is vulnerable. For example, public values change over time and as a consequence it must be understood that was considered acceptable at point A will not necessarily be considered acceptable at point B. In mitigation of this, there may be a role for the previously mentioned citizens’ juries, to measure scientific successes against evolving public values at timely intervals throughout the implementation of the new research norm. In addition, negative scenarios such as data breaches, that could occur even in circumstances of otherwise good governance are highly tractable with the media and can easily become scandals.^12^ These are inconvenient for ensuring a balanced view of the risks and benefits in the mind of the public, and this is a challenge to securing sustained public acceptability for new innovations where matters such as privacy may be at stake.

Notwithstanding challenges such as these, however, it is important to engage in the process according to which the public accept the change in research norms argued for here, in the context of research being done using data platforms. Encouragingly, evidence from the establishment of data platforms such as those to which we have referred—DPUK, MQ Adolescent Mental Health, EHR4CR, All of Us—suggests that they can operate successfully and with trust in their governance of the data to which they provide access. However, these are notable because at present they remain exceptions, and may in this respect be understood as successful proof-of-concept pilot schemes, and in some instances within specific research areas, articulating a vision of how data linkage-driven health research *in general* could be done.

With these considerations and the preceding analysis in mind, we therefore conclude by stating four requirements on which the legitimacy of our proposal rests. First, engagement with the public to explore the tension between the impossibility of providing comprehensive knowledge in advance about the uses to which one’s data might be put, and the probable health gains achievable through those as yet unforeseen uses. Second, a mechanism is required that is sufficiently responsive to individual preferences that they can withdraw consent for future uses of their data if they wish, but only assuming that the reasons for these preferences are not outweighed by public health needs for which data are required. Third, as we have stated, it is incumbent on the institutions handling the data and carrying out the research that they are trustworthy, and ensuring this requires its own governance arrangements, particularly in view of a responsibility to patients and the public. Fourth, honesty is necessary about what the trade-offs are of a revised norm of data reuse and why it should be endorsed and supported as a way of securing important health gains for individuals and society, given the technological climate in which we happen to find ourselves.

## Data Availability

Data sharing not applicable as no datasets generated and/or analysed for this study. There are no data sets associated with this manuscript.

## Footnotes

1 Information about the terms of consent in the All of Us platform can be found in its FAQs: https://www.researchallofus.org/frequently-asked-questions/?_ga=2.75278546.1902005278.1576753949-1286912872.1576753949.

2 The DPUK website provides details of the platform’s governance procedures: https://www.dementiasplatform.uk/about/policies/publication.

3 The EHR4CR website gives an indication of the complexity of consent where research is done across jurisdictions: http://www.ehr4cr.eu/views/resources/faq.cfm#collapse7.

4 The European Data Protection Supervisor has recently acknowledged the assumption that scientific research is good for society, also characterising this as a social contract founded in trust that no irresponsible risks will be taken in the name of this research: https://edps.europa.eu/sites/edp/files/publication/20-01-06_opinion_research_en.pdf Where we talk about a presumption of participation, therefore, we mean in research which has been appropriately scrutinised and does not abuse the social contract.

5 It is worth noting here that there is something odd about the idea of a social contract which one can opt out of. A fundamental part of the social contract idea as it appears in political philosophy is used to justify and account for the idea of state coercion—requiring citizens to pay tax, obey laws, and so on. In this sense, one might expect the social contract idea, as applied in this context, to justify something more like reuse of data without the possibility of opt-out.

6 See R (on the application of W, X, Y and Z) v Secretary of State for Health and Secretary of State for the Home Department, the British Medical Association [2015] EWCA Civ 1034, 44.

7 See Re JR38′s Application for Judicial Review [2015] UKSC 42, [2016] AC 113, 61.

8 See Campbell v Mirror Group Newspapers Ltd [2004] UKHL 22, [2004] All ER 995, 99.

9 https://www.wired.co.uk/article/care-data-nhs-england-closed & https://www.ft.com/content/4ade8884-1b40-11ea-97df-cc63de1d73f4.

10 This condition entails the further question of what type and quantity of evidence would be considered sufficient; however, consideration of the question is beyond the scope and length constraints of this paper.

11 As a matter of law, the expectation of privacy would still be judged in accordance with all the circumstances of the individual case (see note 6).

12 https://www.bbc.co.uk/news/technology-44682369 & https://www.zdnet.com/article/amca-data-breach-has-now-gone-over-the-20-million-mark/.
